# Development and characterization of TrMab-6, a novel anti-TROP2 monoclonal antibody for antigen detection in breast cancer

**DOI:** 10.3892/mmr.2020.11731

**Published:** 2020-11-25

**Authors:** Yusuke Sayama, Mika K. Kaneko, Yukinari Kato

**Affiliations:** 1 Department of Antibody Drug Development, Tohoku University Graduate School of Medicine, Sendai, Miyagi 980-8575, Japan; 2 New Industry Creation Hatchery Center, Tohoku University, Sendai, Miyagi 980-8575, Japan

**Keywords:** TROP2, monoclonal antibody, CBIS, breast cancer

## Abstract

Trophoblast cell-surface antigen 2 (TROP2) is a type I transmembrane glycoprotein that is overexpressed in a number of cancer types, including triple-negative breast cancer. The current study aimed to develop a highly sensitive and specific monoclonal antibody (mAb) targeting TROP2, which could be used to evaluate TROP2 expression using flow cytometry, western blot analysis and immunohistochemistry by employing the Cell-Based Immunization and Screening (CBIS) method. The established anti-TROP2 mAb, TrMab-6 (mouse IgG_2b_, κ), detected TROP2 on PA-tagged TROP2-overexpressing Chinese hamster ovary-K1 (CHO/TROP2-PA) and breast cancer cell lines, including MCF7 and BT-474 using flow cytometry. Western blot analysis indicated a 40 kDa band in lysates prepared from CHO/TROP2-PA, MCF7 and BT-474 cells. Furthermore, TROP2 in 57/61 (93.4%) of the breast cancer specimens was strongly detected using immunohistochemical analysis with TrMab-6. In conclusion, the current study demonstrated that TrMab-6 may be a valuable tool for the detection of TROP2 in a wide variety of breast cancer types.

## Introduction

Breast cancer is by far the most common malignant tumor in women. In 2018, there were 2,088,849 new breast cases (11.6% of the total cancer cases) and 626,679 deaths (6.6% of the total cancer deaths) worldwide (1). Surgery, radiotherapy, chemotherapy, and molecular targeted therapies are currently used for breast cancer treatment; however, effective therapies for patients diagnosed with triple-negative breast cancer [TNBC; i.e., those that are negative for estrogen receptor (ER), progesterone receptor (PR), and human epidermal growth factor receptor 2 (HER2)] remain limited (2–4). TNBC accounts for ~15% of invasive breast cancers; moreover, it tends to be aggressive and is associated with a poor prognosis (2,5,6). TNBC is more common in young women than in older women and is frequently associated with invasion and metastatic disease (2,5–7). As such, highly sensitive and specific monoclonal antibodies (mAbs) are required to facilitate the diagnosis of and treatment decisions for this breast cancer subtype.

The trophoblast cell-surface antigen (TROP2), also known as human tumor-associated calcium signal transducer (TACSTD2), is a type I transmembrane glycoprotein originally identified in human trophoblast cells (8–10). Previously, Schon and Orfanos reported that tunicamycin treatment of living cells and *N*-glycanase digestion of immunopurified TROP2 revealed that the molecular heterogeneity of TROP2 is due to the different *N*-glycosylation in normal and transformed keratinocytes (11). In transformed keratinocytes, two distinct precursor proteins at 38 and 42 kDa were detected, whereas in normal cells the 38-kDa signal was dramatically decreased, indicating that quantitative and qualitative changes of *N*-glycan of TROP2 are associated with the transformation process of human keratinocytes. TROP2 is highly expressed in several cancers and may play a critical role in tumor progression in association with the pathways involving both the extracellular signal-related kinase (ERK) and c-Jun N-terminal kinase (JNK) (12,13). The expression of TROP2 has been reported in more than 85% of all tumors; as such, TROP2 may be a useful marker for cancer diagnosis and immunotherapy (2,14,15). It has also been identified in the stem cells of various tissues, including basal cells, all of which are capable of self-renewal, regeneration, and differentiation (2,16,17). Several mAbs targeting TROP2 are currently evaluated in clinical trials, including PF-06664178 (12,18), IMMU-132 (12,19,20), and DS-1062a (12,21).

In our previous studies, we developed the Cell-Based Immunization and Screening (CBIS) method; in this method, cell lines are used exclusively for both immunization and screening (22). CBIS has been employed to develop sensitive and specific mAbs against numerous transmembrane proteins, including CD19 (23), CD20 (24), CD44 (25), CD133 (22), and PD-L1 (26). Of note, mAbs developed using this method have proven to be extremely useful in flow cytometry, Western blot, and immunohistochemical analyses.

In this study, we developed novel anti-TROP2 mAbs and evaluated their capacity to target breast cancer cells using flow cytometry, Western blot, and immunohistochemical analyses.

## Materials and methods

### 

#### Plasmid preparation

Human TROP2 DNA was synthesized commercially by Thermo Fisher Scientific (Waltham, MA, USA). TROP2 DNA with an N-terminal PA16 tag (27) and a C-terminal RAP tag (28)/MAP tag (29) (PA16-TROP2-RAP-MAP) was subcloned into the pCAG-Ble expression vector (FUJIFILM Wako Pure Chemical Corporation) using an In-Fusion HD Cloning Kit (Takara Bio, Inc.); the recombinant expression vector was named pCAG/PA16-TROP2-RAP-MAP. TROP2 DNA with a C-terminal PA tag (27) alone was also subcloned into the pCAG-Ble vector using an In-Fusion HD Cloning Kit; this expression vector was named pCAG/TROP2-PA. The amino acid sequences of each tag are as follows: PA16 tag, 16 amino acids (GLEGGVAMPGAEDDVV); PA tag, 12 amino acids (GVAMPGAEDDVV); RAP tag, 12 amino acids (DMVNPGLEDRIE); and MAP tag, 12 amino acids (GDGMVPPGIEDK).

#### Cell lines

Chinese hamster ovary (CHO)-K1, P3X63Ag8U.1 (P3U1), BT-474, Lec1, Lec2, and Lec8 cell lines were obtained from the American Type Culture Collection (ATCC; Manassas, VA, USA). MCF7 was obtained from the Cell Resource Center for Biomedical Research, Institute of Development, Aging and Cancer, Tohoku University (Miyagi, Japan).

CHO-K1 cells that overexpress TROP2-PA (CHO/TROP2-PA) and PA16-TROP2-RAP-MAP (CHO/PA16-TROP2-RAP-MAP) were generated by transfection of pCAG/TROP2-PA and pCAG/PA16-TROP2-RAP-MAP to CHO-K1 cells, respectively, using Lipofectamine LTX Reagent (Thermo Fisher Scientific, Inc.). Cell lines Lec1/TROP2, Lec2/TROP2, and Lec8/TROP2 were generated by transfection of pCAG/TROP2-PA to Lec1, Lec2, and Lec8 cells, respectively, using the Neon Transfection System (Thermo Fisher Scientific, Inc.). Several days after the transfection, the transfected cells were confirmed as TROP2-positive by flow cytometry (EC800, Sony Corp.) using a commercial anti-TROP2 antibody (Cat#LS-C489657, LS Bio). The transfected cells were selected by limiting dilution culture and cultivation in the medium containing 0.5 mg/ml of zeocin (InvivoGen). We confirmed the transfection efficiency using western blotting.

The TROP2 gene-deleted cell line, MCF7/TROP2-KO (BINDS-29), was generated by transfection of CRISPR/Cas9 plasmids targeting TROP2 using the Neon Transfection System (Thermo Fisher Scientific, Inc.). Stable transfectants were established by cell sorting using SH800 (Sony Corp.).

CHO-K1, CHO/PA16-TROP2-RAP-MAP, CHO/TROP2-PA, P3U1, MCF7, Lec1/TROP2, Lec2/TROP2, Lec8/TROP2, and BINDS-29 cells were cultured in Roswell Park Memorial Institute (RPMI)-1640 medium (Nacalai Tesque, Inc., Kyoto, Japan); BT-474 cells were cultured in Dulbecco's modified Eagle's medium (DMEM; Nacalai Tesque, Inc.). All media were supplemented with 10% heat-inactivated fetal bovine serum (FBS; Thermo Fisher Scientific, Inc.), 100 U/ml penicillin, 100 µg/ml streptomycin, and 0.25 µg/ml amphotericin B (Nacalai Tesque, Inc.). Cells were grown in an incubator at 37°C with humidity and 5% CO_2_ and 95% air atmosphere.

#### Animals

Female BALB/c mice (6 weeks old) were purchased from CLEA Japan and kept under specific pathogen-free conditions. All animal experiments were conducted in accordance with the relevant guidelines and regulations in order to minimize animal suffering and distress in the laboratory. The Animal Care and Use Committee of Tohoku University approved all the animal experiments (permit no. 2019NiA-001). Mice were euthanized by cervical dislocation under inhalation anesthesia using 2% of isoflurane, and the death was verified to be respiratory and cardiac arrest.

#### Hybridoma production

We employed CBIS to develop new mAbs against TROP2. Two mice were immunized with CHO/PA16-TROP2-RAP-MAP cells (1×10^8^) *via* the intraperitoneal route (i.p.) together with the Imject Alum (Thermo Fisher Scientific, Inc.). After several additional immunizations, a booster immunization was administered *via* the i.p. route 2 days before spleen cell collection. Mice were euthanized by cervical dislocation under inhalation anesthesia using isoflurane, and the death was verified to be respiratory and cardiac arrest. We chopped spleens, and collected spleen cells using serum-free RPIM-1640 medium. We further broke the red blood cells with 3 ml of Red Blood Cell Lysing Buffer Hybri-Max (Sigma-Aldrich Corp.) at 37°C for 1 min, and washed the spleen cells using serum-free RPIM-1640 medium. The collected spleen cells were fused with P3U1 mouse myeloma cells using polyethylene glycol 1500 (Roche Diagnostics) (30,31); the resulting hybridomas were selected in RPMI medium, including hypoxanthine, aminopterin, and thymidine (Thermo Fisher Scientific, Inc.). The culture supernatants were screened *via* flow cytometry using CHO/TROP2-PA and CHO-K1 cells.

#### Flow cytometry

Cells were collected following a brief exposure to 0.25% trypsin and 1 mM ethylenediaminetetraacetic acid (EDTA; Nacalai Tesque, Inc.). The cells were washed with 0.1% bovine serum albumin in phosphate-buffered saline (PBS) and treated with anti-TROP2 mAbs, such as TrMab-6 (1 µg/ml) or EPR20043 (1/60 dilution; Abcam) for 30 min at 4°C. After incubation, the cells were treated with Alexa Fluor 488-conjugated anti-mouse IgG (1:1,000; Cell Signaling Technology, Inc.) or Alexa Fluor 488-conjugated anti-rabbit IgG (1:1,000; Cell Signaling Technology, Inc.). Fluorescence data were collected using SA3800 Spectral Cell Analyzer (Sony Corp.) and analyzed using FlowJo (BD Biosciences).

#### Determination of the binding affinity

MCF7 or BT-474 cells (2×10^5^) were suspended in 100 µg of serially diluted TrMab-6 (6 ng/ml-100 µg/ml) for 30 min at 4°C, followed by the addition of Alexa Flour 488-conjugated anti-mouse IgG (1:200; Cell Signaling Technologies, Inc.). Fluorescence data were collected using a cell analyzer (EC800). The dissociation constant (*K*_D_) was calculated by fitting the binding isotherms to built-in, one-site binding models in GraphPad Prism 8 (GraphPad Software, Inc.).

#### Western blot analysis

Cell lysates (10 µg) were boiled in sodium dodecyl sulfate (SDS) sample buffer (Nacalai Tesque, Inc.). Proteins were separated on 5–20% polyacrylamide gels (FUJIFILM Wako Pure Chemical Corporation) and transferred onto polyvinylidene difluoride (PVDF) membranes (Merck KGaA). After blocking with 4% skim milk (Nacalai Tesque, Inc.) in PBS with 0.05% Tween-20, the membranes were incubated with 1 or 5 µg/ml of TrMab-6, 1/2000 dilution of EPR20043 (Abcam), 1 µg/ml of NZ-1 (anti-PA tag), or 1 µg/ml of anti-β-actin (clone AC-15; Sigma-Aldrich Corp.). This was followed by incubation with peroxidase-conjugated anti-mouse immunoglobulins (Agilent Technologies Inc.; diluted 1:1,000) to detect TrMab-6 and anti-β-actin, peroxidase-conjugated anti-rabbit immunoglobulins (Agilent Technologies Inc.; diluted 1:1,000) to detect EPR20043, or anti-rat IgG (Sigma-Aldrich Corp; diluted 1:10,000) to detect NZ-1, respectively. Finally, protein bands were detected with ImmunoStar LD (FUJIFILM Wako Pure Chemical Corporation) using a Sayaca-Imager (DRC Co. Ltd.).

#### Immunohistochemical analysis

Paraffin-embedded tissue sections of the breast cancer tissue array (Cat#T8235721-5, Lot#B104066; BioChain, San Francisco, CA, USA) were autoclaved in EnVision FLEX Target Retrieval Solution High pH (Agilent Technologies, Inc.) for 20 min. After blocking with SuperBlock T20 (Thermo Fisher Scientific, Inc.), tissue sections were incubated with TrMab-6 (5 µg/ml) or EPR20043 (1/500 dilution; Abcam) for 1 h at room temperature and then treated with the EnVision+ Kit for mouse (Agilent Technologies Inc.) and EnVision+ Kit for rabbit (Agilent Technologies Inc.) for 30 min, respectively. Color was developed using 3,3′-diaminobenzidine tetrahydrochloride (DAB; Agilent Technologies Inc.) for 2 min. Counterstaining was performed with hematoxylin (FUJIFILM Wako Pure Chemical Corporation).

## Results

### 

#### Development of novel anti-TROP2 mAbs using the CBIS method

**Figure 1.**
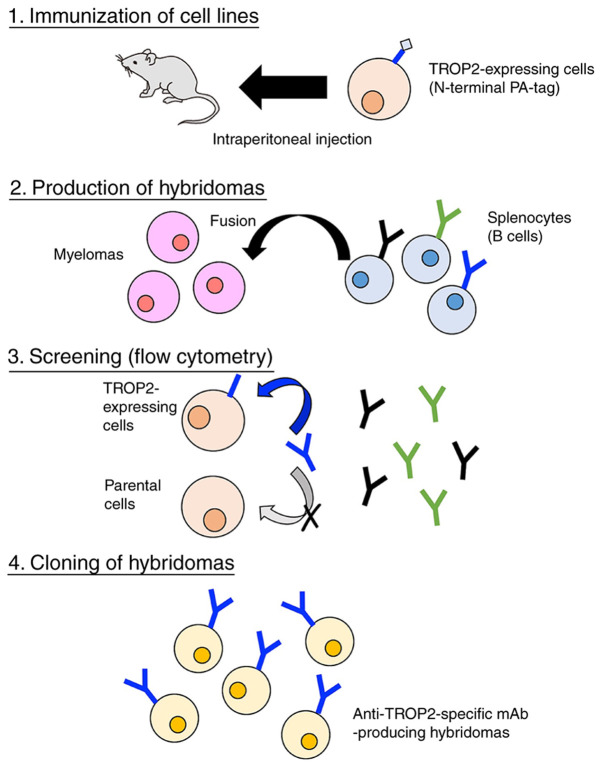
Illustration of Cell-Based Immunization and Screening. A total of two mice were immunized with CHO/PA16-TROP2-RAP-MAP cells, and anti-TROP2 monoclonal antibodies were screened using flow cytometry. TROP2, TROP2, trophoblast cell-surface antigen 2.

We immunized two mice with CHO/PA16-TROP2- RAP-MAP cells and anti-TROP2 mAbs were screened *via* flow cytometry (Fig. 1). The first screening approach identified strong signals from CHO/TROP2-PA cells and weak to no signals from CHO-K1 cells using hybridoma supernatants from 90 of the 956 wells (9.4%). The second screening approach identified strong signals from MCF7 cells from 84 of the 90 hybridoma supernatants identified in the earlier step (93.3%). After limiting dilution, we established 30 positive clones. Further screening *via* Western blot and immunohistochemistry led to the establishment of TrMab-6. The subclass of TrMab-6 was determined to be mouse IgG_2b_ as shown in Fig. S1A.

#### Flow cytometry analysis

**Figure 2.**
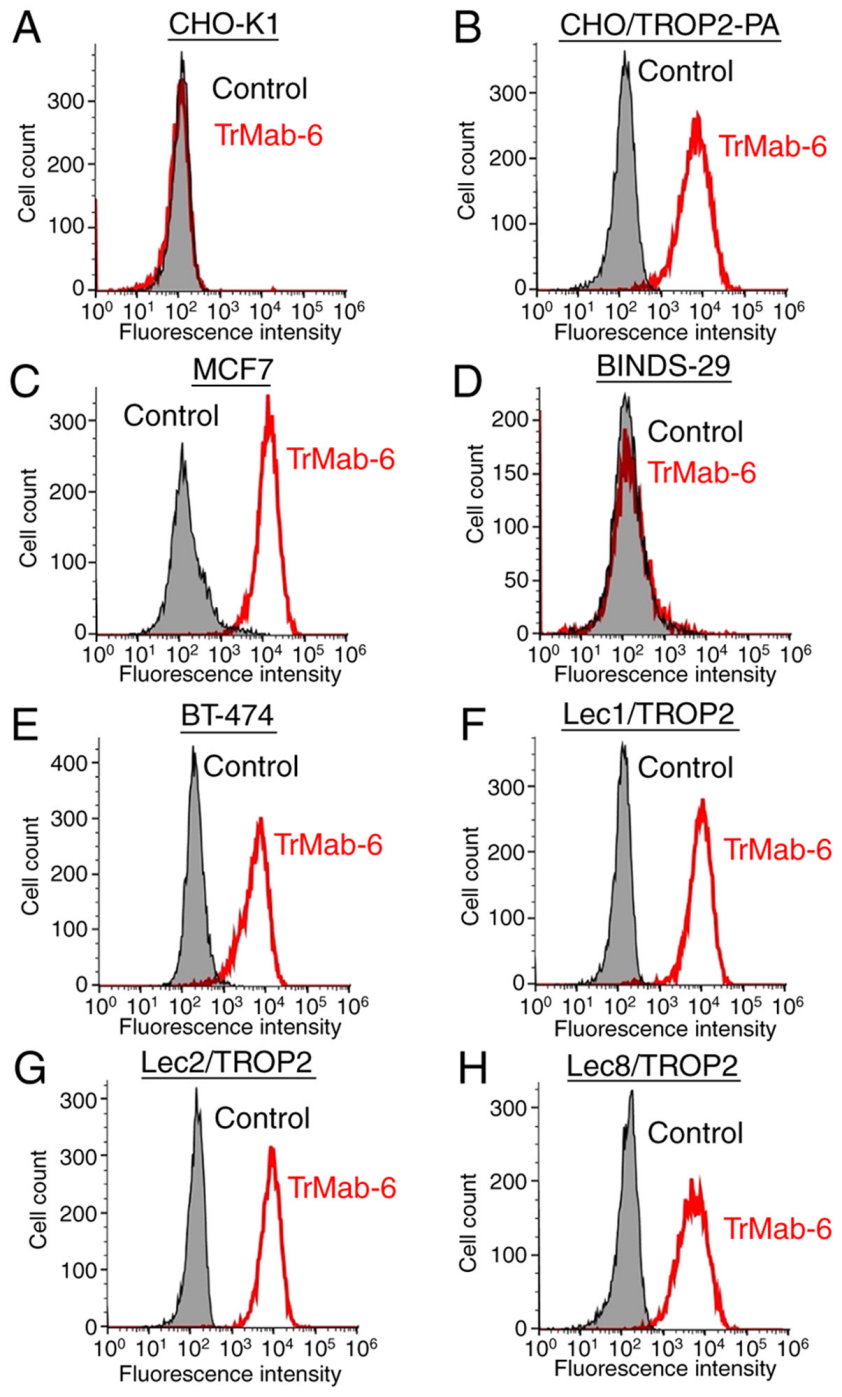
Flow cytometric detection of TROP2 using TrMab-6. (A) CHO-K1, (B) CHO/TROP2-PA, (C) MCF7, (D) BINDS-29 (MCF7/TROP2-KO), (E) BT-474, (F) Lec1/TROP2, (G) Lec2/TROP2 and (H) Lec8/TROP2 cells were incubated with TrMab-6 (1 µg/ml; red line) or 0.1% BSA in PBS (grey) for 30 min, followed by Alexa Fluor 488-conjugated secondary antibodies. Fluorescence data were collected using a cell analyzer. TROP2, trophoblast cell-surface antigen 2.

We developed several transfectants, such as CHO/TROP2-PA, Lec1/TROP2, Lec2/TROP2, and Lec8/TROP2, and the transfection efficiency was confirmed using an anti-PA tag mAb (NZ-1) by Western blot analysis (Fig. S1B). Then, we performed flow cytometry targeting several relevant cell lines in order to characterize antigen detection using TrMab-6 (Fig. 2). TrMab-6 detected CHO/TROP2-PA cells, but not parental CHO-K1 cells. TrMab-6 also detected endogenous TROP2 on human breast cancer cell lines, including MCF7 and BT-474. Contrarily, TrMab-6 did not react with BINDS-29 (TROP2-gene-deleted MCF7 cells). Taken together, these results suggested that TrMab-6 is specific for TROP2. As shown in Fig. S2, another anti-TROP2 mAb (clone EPR20043) weakly reacted with MCF7, but did not react with BT-474 although TrMab-6 strongly reacted with both MCF7 and BT-474, indicating that TrMab-6 is more useful for flow cytometry than EPR20043 although EPR20043 was shown to be useful in all applications, such as flow cytometry, Western blot, and immunohistochemical analyses (Table SI).

Next, we investigated whether the epitope of TrMab-6 is associated with glycans. Thus, we performed flow cytometry using TROP2-transfected glycan-deficient CHO cells, including those deficient in Lec1 (*N*-glycan-deficient), Lec2 (sialic acid-deficient), and Lec8 (galactose-deficient) cells. As presented in Fig. 2, TrMab-6 reacted with Lec1/TROP2, Lec2/TROP2, and Lec8/TROP2 cells to an extent indistinguishable from that observed with CHO/TROP2-PA. These results indicated that the binding epitope recognized by TrMab-6 was unlikely to be associated with glycans.

#### Determination of the binding affinity using TrMab-6 against breast cancers by flow cytometry

**Figure 3.**
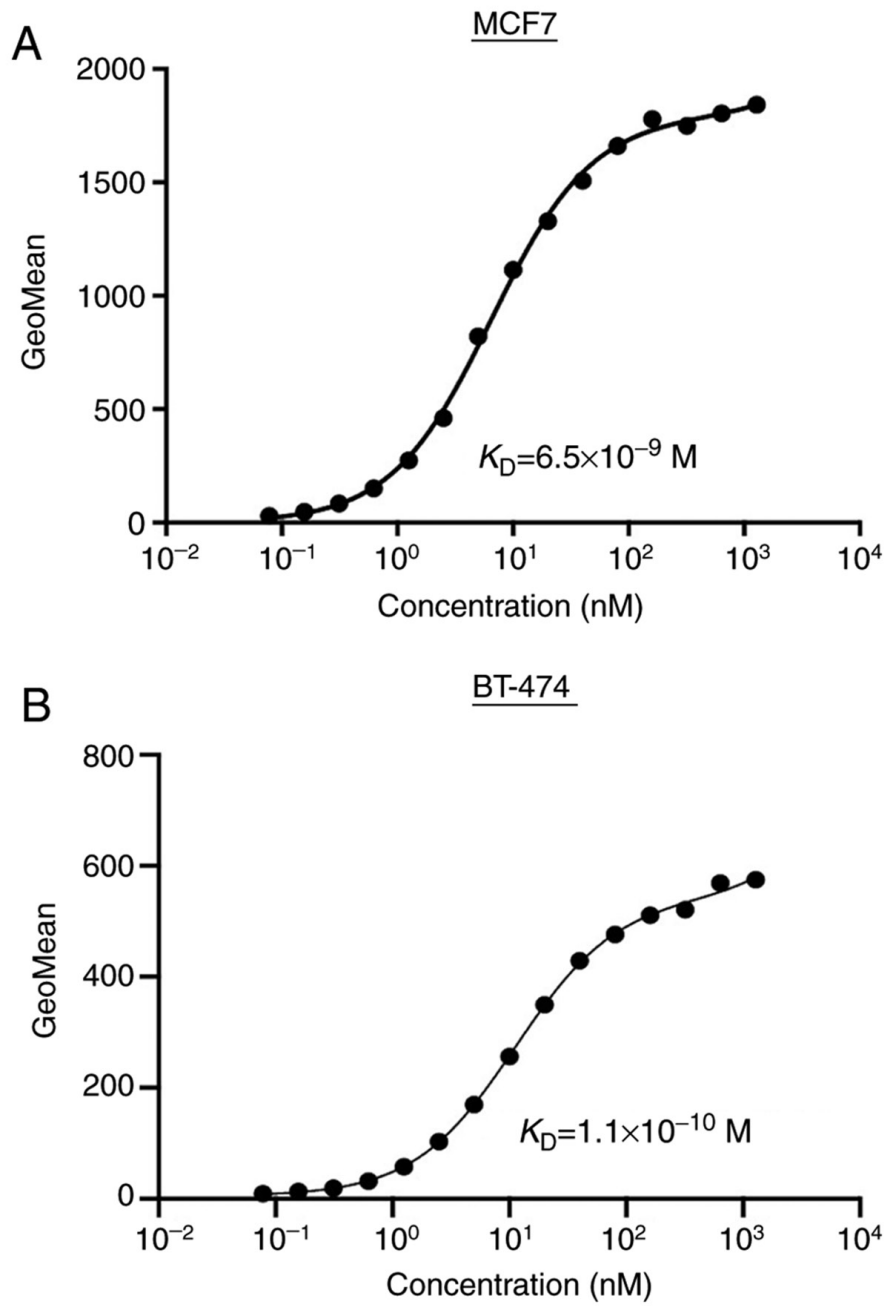
Flow cytometric determination of the binding affinity of TrMab-6 for breast cancer cells. (A) MCF7 or (B) BT-474 cells were suspended in 100 ul of serially diluted antibodies (6 ng/ml to 100 µg/ml), followed by incubation with Alexa Fluor 488-conjugated secondary antibodies. Fluorescence data were collected using a cell analyzer; GeoMean, geometric mean of fluorescence intensity.

To determine the binding affinity of TrMab-6, we conducted kinetic analysis of the interaction of TrMab-6 with MCF7 and BT-474 cells *via* flow cytometry. The *K*_D_ of TrMab-6 was determined to be 6.5×10^−9^ M when targeting MCF7 cells and 1.1×10^−10^ M for BT-474 cells (Fig. 3). These results indicated that TrMab-6 binds with high affinity to TROP2-expressing breast cancer cells.

#### Western blot analyses

**Figure 4.**
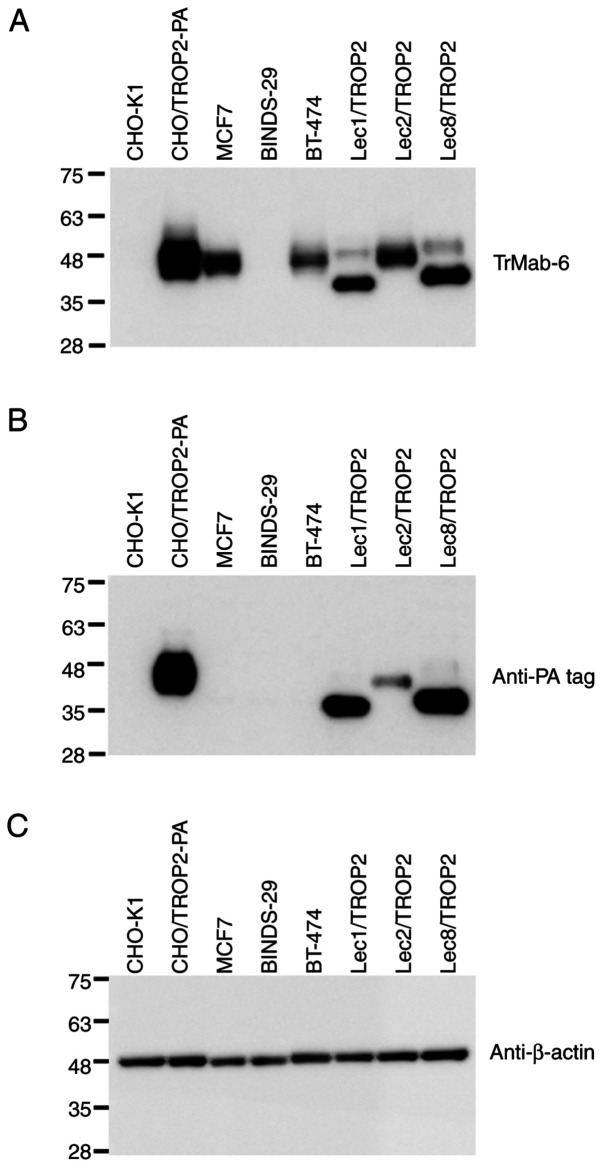
Detection of TROP2 with TrMab-6 using western blot analysis. The cell lysates (10 µg) of CHO-K1, CHO/TROP2-PA, MCF7, BINDS-29 (MCF7/TROP2-KO), BT-474, Lec1/TROP2, Lec2/TROP2 and Lec8/TROP2 were subjected to SDS-PAGE and transferred onto the PVDF membranes. The membranes were incubated with l µg/ml of (A) TrMab-6, an (B) anti-PA mAb (NZ-1) and an (C) anti-β-actin mAb (AC-15), followed by secondary antibodies. TROP2, trophoblast cell-surface antigen 2.

TrMab-6 binding identified TROP2 as an immunoreactive band with an estimated 40 kDa band in lysates prepared from CHO/TROP2-PA, MCF7, and BT-474 cells; no immunoreactive bands were found in CHO-K1 and TROP2-gene-deleted MCF7 (BINDS-29) cells (Fig. 4), again confirming its specificity for TROP2. TrMab-6 also detected TROP2 of Lec1/TROP2, Lec2/TROP2, and Lec8/TROP2. Although TROP2 proteins, which were expressed in Lec1/TROP2 and Lec8/TROP2, were detected in lower molecular weight compared with CHO/TROP2-PA and Lec2/TROP2, the intensity by TrMab-6 was similar among those cell lines, indicating that the binding epitope of TrMab-6 is independent of glycans. An anti-PA tag mAb (NZ-1) also detected TROP2 bands in lysates of CHO/TROP2-PA, Lec1/TROP2, Lec2/TROP2, and Lec8/TROP2 cells. These results indicated that TrMab-6 could be used to detect TROP2 expressed by breast cancer cells *via* Western blot.

We compared the reactivity of TrMab-6 and another anti-TROP2 mAb (clone EPR20043) in Western blot analysis. As shown in Fig. S3, both TrMab-6 and EPR20043 strongly detected TROP2 from both MCF7 and BT-474, indicating that both TrMab-6 and EPR20043 are useful for Western blot analysis.

#### Immunohistochemical analyses against breast cancer

**Figure 5.**
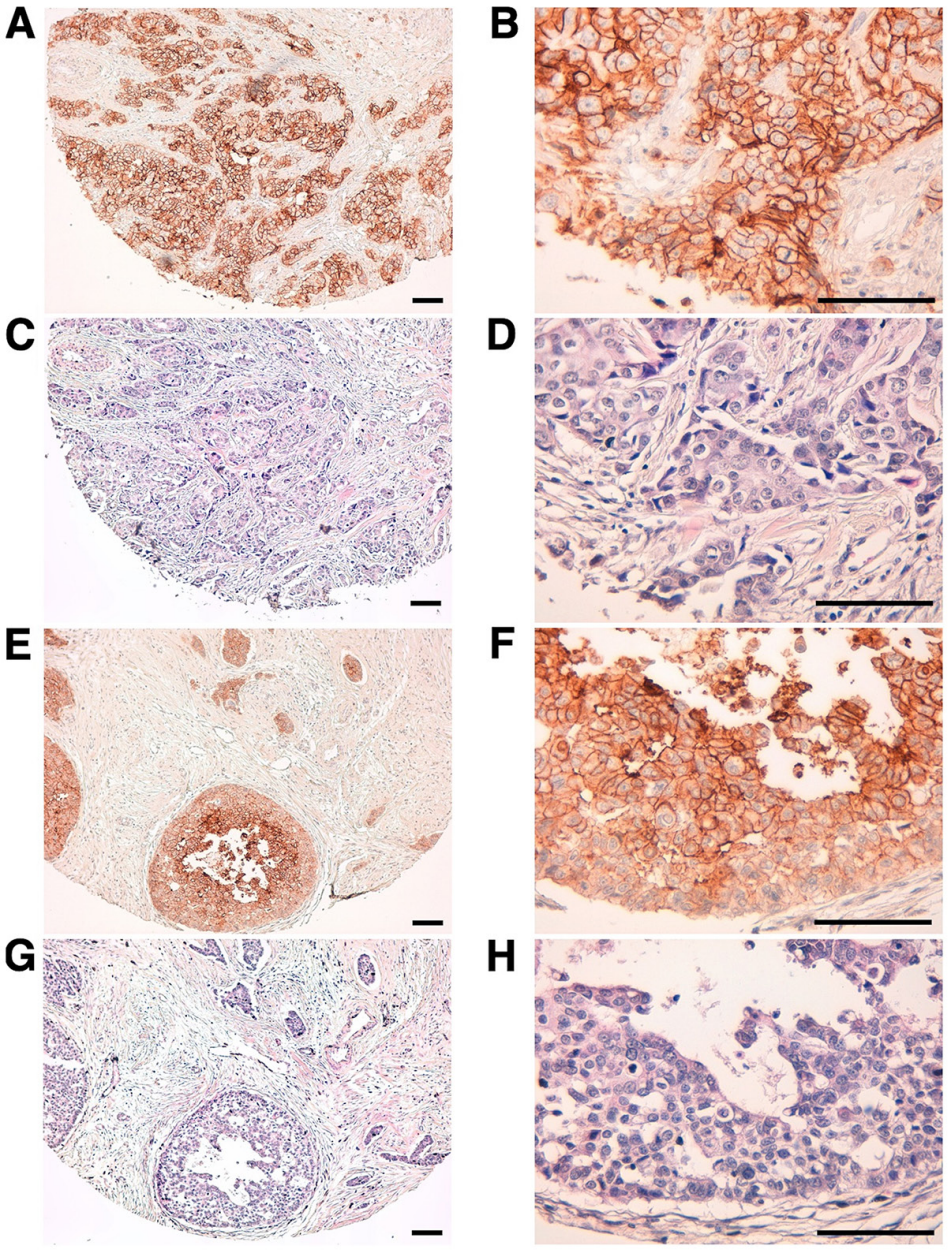
Detection of TROP2 in breast cancer specimens using immunohistochemical analysis with TrMab-6. (A and B) Tissue sections of patients with human breast cancer (invasive ductal carcinoma, Case No. 5) were incubated with 5 µg/ml of TrMab-6 and then treated with the EnVision+ kit. Tissues were counterstained with hematoxylin. (C and D) H&E staining was performed using consecutive breast cancer tissues (invasive ductal carcinoma, Case No. 14). (E and F) Tissue sections of patients with human breast cancer (invasive lobular carcinoma) were incubated with 5 µg/ml of TrMab-6 and treated with a EnVision+ kit. Counterstaining was performed with hematoxylin. (G and H) H&E staining was performed using consecutive breast cancer tissues (invasive lobular carcinoma). Scale bar, 100 µm. TROP2, trophoblast cell-surface antigen 2; H&E, hematoxylin and eosin.

We then used TrMab-6 to target clinical specimens of human breast cancer tissue *via* immunohistochemical analysis (Table I). TrMab-6 detected TROP2 in 57/61 of the breast cancer specimens (93.4%; Table II). Among these specimens were 50/54 cases (93.1%) of invasive ductal carcinoma (Table II). Typical TrMab-6-associated staining patterns in the specimens of invasive ductal carcinomas are presented in Fig. 5A and B. Hematoxylin and eosin (H&E) staining of invasive ductal carcinoma tissue is presented in Fig. 5C and D. Furthermore, TrMab-6 detected TROP2 in 4/4 cases (100%) of invasive lobular carcinoma, 2/2 cases (100%) of adenocarcinoma, and 1/1 case (100%) of medullary carcinoma (Table II); the typical staining patterns of invasive lobular carcinoma are presented in Fig. 5E and F, and H&E staining was performed as presented in Fig. 5G and H. Among the 61 breast cancer cases, 30/61 cases (49.2%) were stained strongly positive, 18/61 cases (29.5%) were stained moderately positive, and 9/61 cases (14.8%) were stained weakly positive by TrMab-6 (Table II). We obtained the information about ER, PR, and HER2 (Table I). To determine HER2 expression, we used an anti-HER2 mAb (clone H_2_Mab-77) (32). Among 61 breast cancers, 31 cases (50.8%) were determined to be triple-negative (Table III). Interestingly, 28/31 (90.3%) were stained by TrMab-6; especially, 17/31 (54.8%) were stained strongly positive by TrMab-6 (Table III), indicating that triple-negative breast cancers should be an ideal target of anti-TROP2 mAbs, including TrMab-6.

We compared the reactivity of TrMab-6 and another anti-TROP2 mAb (clone EPR20043) in immunohistochemical analysis. As shown in Table I, EPR20043 stained 60/61 (98.4%) breast cancer tissues, although TrMab-6 stained 57/61 (93.4%) breast cancer tissues, indicating that EPR20043 is more useful for immunohistochemical analysis than TrMab-6.

## Discussion

Generating a mAb that can be utilized for multiple applications, including flow cytometry, Western blot, and immunohistochemistry, is usually difficult. Using the CBIS method, in which antigen-expressing cell lines are used for both immunization and screening (22), we have developed numerous useful mAbs that target membrane proteins, including CD19 (23), CD20 (24), CD44 (25), CD133 (22), PD-L1 (26), and podoplanin (PDPN) (33–36). Among these unique targets, CD20 has four membrane-spanning domains and only two small extracellular domains that include amino acids 72–80 and 142–182 (37,38). Although there are several commercially available mAbs that interact with amino acids 142–182 of CD20 and are specifically useful in flow cytometry, there are no available anti-CD20 mAbs that are effective not only in flow cytometry but also in Western blot and immunohistochemical analyses. We recently developed clone C_20_Mab-11, which can detect CD20 associated with B-cell lymphoma by flow cytometry, Western blot, and immunohistochemical analyses (24).

Likewise, we herein aimed to establish one or more multipurpose anti-TROP2 mAbs because the applications of commercially available anti-TROP2 mAbs were somewhat limited (Table SI). Using the CBIS method, we successfully developed a sensitive and specific novel anti-TROP2 mAb (clone TrMab-6) that can be used in every application, including flow cytometry (Fig. 3), Western blot (Fig. 4), and immunohistochemical analyses (Fig. 5). Another anti-TROP2 mAb (clone EPR20043 from Abcam) is more sensitive than TrMab-6 in immunohistochemical analysis (Table I), but not useful in flow cytometry (Fig. S2). EPR20043 might react with intracellular region of TROP2 although the immunogen was not clearly shown in its application sheet. Clone SP293 (Abcam) was also shown to be useful for flow cytometry, Western blot, and immunohistochemical analyses in its application sheet; however, the intracellular region of TROP2 was also used as immunogen (Table SI).

We conclude that TrMab-6 is more advantageous than the other anti-TROP2 mAbs, such as EPR20043 and SP293 because TrMab-6 is useful to detect TROP2 in all applications, such as flow cytometry in non-fixed condition, Western blot, and immunohistochemical analysis. However, in this study, TrMab-6 was shown to be useful for only *in vitro* experiments. In the future, we will determine whether TrMab-6 would be suitable for use as targeted molecular therapy against breast cancers. The subclasses of mouse IgG, IgG_2a_ and IgG_2b_, both induce antibody-dependent cellular cytotoxicity (ADCC) or complement-dependent cytotoxicity (CDC) (39,40). TrMab-6 was determined to be of the mouse IgG_2b_ subclass (Fig. S1). Although we performed ADCC reporter assay using TrMab-6, ADCC activity was not observed unexpectedly (data not shown). To promote antibody therapy using TrMab-6, we will utilize antibody-drug conjugates (ADCs), radioimmunotherapy (RIT), photoimmunotherapy (PIT), or chimeric antigen receptor T-cell (CAR-T) therapy. About ADCs targeting TROP2, conjugation of the irinotecan metabolite, SN-38, to a humanized anti-TROP2 antibody (sacituzumab govitecan) promotes broad and potent antitumor effects in human cancer xenografts and in patients with advanced triple-negative breast, non-small-cell and small-cell lung, and urothelial cancers (14). About RIT, van Rij *et al* (41) previously reported that TROP2-expressing prostate cancer can be targeted efficiently with TF12 [anti-TROP2 × anti-HSG (histamine-succinyl-glycine)] and ^177^Lu-labeled diHSG-peptide (IMP288). Furthermore, Nishimura *et al* (42) selected TROP2 as a molecular target for PIT, and utilized a newly developed humanized anti-TROP2 mAb conjugated to the photosensitizer IR700 (TROP2-IR700) for the treatment of pancreatic carcinoma and cholangiocarcinoma. Growth of tumor xenografts was significantly inhibited in response to TROP2-targeted PIT relative to controls, suggesting that TROP2-targeted PIT is also an important means for improving treatment for TROP2-expressing cancers. About CAR-T, Zhao *et al* (43) reported that novel bi-specific TROP2/PD-L1 CAR-T cells could target TROP2/PD-L1 and checkpoint blockade, resulting in cytotoxicity for gastric cancer cells. These results also suggested that CAR-T cell therapy featuring TROP2 can be developed to target TROP2-expressing cancers. These various modalities will allow us to explore TrMab-6-mediated antitumor activities in the mouse xenograft model of breast cancer.

## Supplementary Material

Supporting Data

## Figures and Tables

**Table I.** Results of TrMab-6 and EPR20043 immunostaining in 61 breast cancers.

Case	Age	Sex	Pathological diagnosis	Differentiation	TNM	ER	PR	HER2/H_2_Mab-77	TrMab-6	EPR20043
1	44	F	Invasive ductal carcinoma	Moderately	T2 N2 M1	–	–	–	3+	3+
2	58	F	Medullary carcinoma	Moderately	T2 N2 M1	–	–	–	3+	2+
3	40	F	Invasive ductal carcinoma	Moderately	T2 N1 M0	1+	–	1+	2+	3+
4	52	F	Invasive ductal carcinoma	Moderately	T2 N2 M1	1+	1+	–	1+	2+
5	60	F	Invasive ductal carcinoma	Moderately	T2 N1 M1	–	–	–	3+	3+
6	57	F	Invasive ductal carcinoma	Moderately	T2 N0 M0	–	–	–	2+	3+
7	48	F	Invasive ductal carcinoma	Moderately	T2 N0 M0	1+	2+	2+	2+	3+
8	66	F	Invasive lobular carcinoma	Moderately	T2 N0 M0	2+	1+	–	2+	2+
9	58	F	Adenocarcinoma	Moderately	T2 N2 M1	1+	–	–	3+	3+
10	63	F	Invasive ductal carcinoma	Moderately	T2 N0 M0	3+	3+	–	3+	3+
11	32	F	Invasive ductal carcinoma	Moderately	T2 N0 M0	–	–	–	2+	3+
12	59	F	Invasive lobular carcinoma	Well	T2 N2 M0	–	–	–	1+	1+
13	44	F	Invasive lobular carcinoma	Well	T2 N2 M0	1+	1+	1+	2+	3+
14	60	F	Invasive lobular carcinoma	Moderately	T2 N1 M0	3+	2+	–	3+	3+
15	44	F	Invasive ductal carcinoma	Moderately	T2 N2 M0	–	3+	3+	3+	3+
16	82	F	Invasive ductal carcinoma	Moderately	T2 N1 M1	–	–	–	3+	3+
17	58	F	Adenocarcinoma	Moderately	T2 N1 M1	–	–	1+	3+	3+
18	57	F	Invasive ductal carcinoma	Well	T3 N3 M0	2+	1+	–	3+	3+
19	41	F	Invasive ductal carcinoma	Moderately	T2 N1 M0	–	–	–	3+	3+
20	44	F	Invasive ductal carcinoma	Moderately	T2 N2 M0	–	–	–	3+	1+
21	78	F	Invasive ductal carcinoma	Moderately	T2 N1 M0	2+	1+	–	3+	3+
22	60	F	Invasive ductal carcinoma	Moderately	T2 N0 M0	–	–	1+	1+	1+
23	N/A	F	Invasive ductal carcinoma	Moderately	T2 N1 M1	3+	–	2+	3+	2+
24	46	F	Invasive ductal carcinoma	Moderately	T2 N3 M1	–	–	–	3+	3+
25	41	F	Invasive ductal carcinoma	Moderately	T2 N2 M0	–	–	–	3+	3+
26	59	F	Invasive ductal carcinoma	Poorly	T2 N0 M0	2+	1+	–	2+	2+
27	45	F	Invasive ductal carcinoma	Poorly	T2 N0 M0	1+	1+	–	–	1+
28	43	F	Invasive ductal carcinoma	N/A	T2 N1 M1	–	–	–	1+	1+
29	40	F	Invasive ductal carcinoma	N/A	T1 N0 M0	1+	1+	–	2+	1+
30	51	F	Invasive ductal carcinoma	Moderately	T2 N2 M0	1+	1+	–	2+	3+
31	45	F	Invasive ductal carcinoma	Poorly	T2 N0 M0	1+	2+	1+	2+	3+
32	45	F	Invasive ductal carcinoma	Poorly	T2 N1 M0	2+	3+	3+	3+	3+
33	47	F	Invasive ductal carcinoma	Moderately-Poorly	T2 N1 M0	–	–	–	3+	3+
34	55	F	Invasive ductal carcinoma	Moderately	T2 N3 M1	–	–	1+	3+	3+
35	58	F	Invasive ductal carcinoma	Moderately	T3 N3 M0	1+	1+	–	2+	3+
36	47	F	Invasive ductal carcinoma	Moderately	T2 N0 M0	–	–	–	1+	2+
37	38	F	Invasive ductal carcinoma	Poorly	T2 N0 M0	–	–	–	3+	3+
38	40	F	Invasive ductal carcinoma	Poorly	T2 N0 M0	–	–	–	3+	3+
39	57	F	Invasive ductal carcinoma	Poorly	T2 N0 M0	–	–	–	1+	1+
40	42	F	Invasive ductal carcinoma	Moderately	T2 N0 M0	–	–	2+	3+	3+
41	60	F	Invasive ductal carcinoma	Moderately	T2 N0 M0	–	–	–	3+	3+
42	58	F	Invasive ductal carcinoma	Moderately	T2 N0 M0	–	1+	–	1+	1+
43	41	F	Invasive ductal carcinoma	Moderately	T2 N0 M0	–	3+	–	2+	2+
44	50	F	Invasive ductal carcinoma	Moderately	T2 N0 M0	–	–	–	3+	3+
45	60	F	Invasive ductal carcinoma	Moderately	T2 N2 M1	1+	1+	–	3+	2+
46	53	F	Invasive ductal carcinoma	Moderately	T2 N0 M0	–	–	–	3+	3+
47	65	F	Invasive ductal carcinoma	Moderately	T2 N0 M0	–	–	–	1+	2+
48	43	F	Invasive ductal carcinoma	Moderately	T2 N0 M0	–	–	–	3+	3+
49	57	F	Invasive ductal carcinoma	Moderately	T2 N0 M0	–	–	3+	3+	3+
50	37	F	Invasive ductal carcinoma	Moderately	T2 N0 M0	–	1+	–	2+	3+
51	50	F	Invasive ductal carcinoma	Moderately	T2 N3 M0	–	–	–	–	2+
52	48	F	Invasive ductal carcinoma	Poorly	T2 N1 M0	–	–	–	1+	1+
53	50	F	Invasive ductal carcinoma	Moderately	T2 N0 M0	–	–	–	3+	3+
54	53	F	Invasive ductal carcinoma	Moderately	T2 N0 M0	–	–	–	3+	3+
55	49	F	Invasive ductal carcinoma	Moderately	T2 N0 M0	–	–	–	2+	2+
56	65	F	Invasive ductal carcinoma	Moderately	T2 N1 M0	1+	1+	–	2+	3+
57	43	F	Invasive ductal carcinoma	Moderately	T2 N0 M0	–	–	–	–	–
58	58	F	Invasive ductal carcinoma	Moderately	T2 N0 M0	–	–	–	–	1+
59	48	F	Invasive ductal carcinoma	Moderately	T2 N0 M0	–	–	–	2+	3+
60	N/A	F	Invasive ductal carcinoma	Moderately	N/A	–	–	–	2+	2+
61	N/A	F	Invasive ductal carcinoma	Moderately-Poorly	N/A	1+	1+	–	2+	3+

N/A, not available; TNM, tumor node metastasis; F, female; ER, estrogen receptor; PR, progesterone receptor.

**Table II.** Results of TrMab-6 immunostaining in 61 breast cancers.

		TrMab-6				
			
Pathological diagnosis	No. of cases	3+	2+	1+	−	No. of positive cases (%)
Invasive ductal carcinoma	54	26	16	8	4	50/54 (92.6)
Invasive lobular carcinoma	4	1	2	1	0	4/4 (100)
Adenocarcinoma	2	2	0	0	0	2/2 (100)
Medullary carcinoma	1	1	0	0	0	1/1 (100)
Total	61	30/61 (49.2%)	18/61 (29.5%)	9/61 (14.8%)	4/61 (6.6%)	57/61 (93.4%)

**Table III.** Results of TrMab-6 immunostaining in 31 triple negative breast cancers.

		TrMab-6				
			
Breast cancer subtype	No. of cases	3+ (%)	2+ (%)	1+ (%)	- (%)	No. of positive cases
Triple negative breast cancer	31	17 (54.8)	5 (16.1)	6 (19.4)	3 (9.7)	28/31 (90.3%)

## Data Availability

The datasets used and/or analyzed during the current study are available from the corresponding author on reasonable request.

## Abbreviations

ADCC: antibody-dependent cellular cytotoxicity
CBIS: Cell-Based Immunization and Screening
CHO: Chinese hamster ovary
DMEM: Dulbecco's modified Eagle's medium
EDTA: ethylenediaminetetraacetic acid
mAb: monoclonal antibody
H&E: hematoxylin and eosin
RPMI: Roswell Park Memorial Institute
TACTD2: tumor-associated calcium signal transducer 2
TROP2: trophoblast cell-surface antigen 2

## References

[1] Bray F, Ferlay J, Soerjomataram I, Siegel RL, Torre LA, Jemal A (2018). Global cancer statistics 2018: GLOBOCAN estimates of incidence and mortality worldwide for 36 cancers in 185 countries. CA Cancer J Clin.

[2] Bardia A, Mayer IA, Vahdat LT, Tolaney SM, Isakoff SJ, Diamond JR, O'Shaughnessy J, Moroose RL, Santin AD, Abramson VG (2019). Sacituzumab govitecan-hziy in refractory metastatic triple-negative breast cancer. N Engl J Med.

[3] Anders CK, Zagar TM, Carey LA (2013). The management of early-stage and metastatic triple-negative breast cancer: A review. Hematol Oncol Clin North Am.

[4] Trivers KF, Lund MJ, Porter PL, Liff JM, Flagg EW, Coates RJ, Eley JW (2009). The epidemiology of triple-negative breast cancer, including race. Cancer Causes Control.

[5] Plasilova ML, Hayse B, Killelea BK, Horowitz NR, Chagpar AB, Lannin DR (2016). Features of triple-negative breast cancer: Analysis of 38,813 cases from the national cancer database. Medicine (Baltimore).

[6] Kohler BA, Sherman RL, Howlader N, Jemal A, Ryerson AB, Henry KA, Boscoe FP, Cronin KA, Lake A, Noone AM (2015). Annual report to the nation on the status of cancer, 1975–2011, featuring incidence of breast cancer subtypes by race/ethnicity, poverty, and state. J Natl Cancer Inst.

[7] DeSantis CE, Fedewa SA, Goding Sauer A, Kramer JL, Smith RA, Jemal A (2016). Breast cancer statistics, 2015: Convergence of incidence rates between black and white women. CA Cancer J Clin.

[8] Fornaro M, Dell'Arciprete R, Stella M, Bucci C, Nutini M, Capri MG, Alberti S (1995). Cloning of the gene encoding Trop-2, a cell-surface glycoprotein expressed by human carcinomas. Int J Cancer.

[9] Alberti S, Miotti S, Stella M, Klein CE, Fornaro M, Menard S, Colnaghi MI (1992). Biochemical characterization of Trop-2, a cell surface molecule expressed by human carcinomas: Formal proof that the monoclonal antibodies T16 and MOv-16 recognize Trop-2. Hybridoma.

[10] Lipinski M, Parks DR, Rouse RV, Herzenberg LA (1981). Human trophoblast cell-surface antigens defined by monoclonal antibodies. Proc Natl Acad Sci USA.

[11] Schon MP, Orfanos CE (1995). Transformation of human keratinocytes is characterized by quantitative and qualitative alterations of the T-16 antigen (Trop-2, MOv-16). Int J Cancer.

[12] Zaman S, Jadid H, Denson AC, Gray JE (2019). Targeting Trop-2 in solid tumors: Future prospects. Onco Targets Ther.

[13] Guan H, Guo Z, Liang W, Li H, Wei G, Xu L, Xiao H, Li Y (2017). Trop2 enhances invasion of thyroid cancer by inducing MMP2 through ERK and JNK pathways. BMC Cancer.

[14] Goldenberg DM, Stein R, Sharkey RM (2018). The emergence of trophoblast cell-surface antigen 2 (TROP-2) as a novel cancer target. Oncotarget.

[15] Goldenberg DM, Cardillo TM, Govindan SV, Rossi EA, Sharkey RM (2015). Trop-2 is a novel target for solid cancer therapy with sacituzumab govitecan (IMMU-132), an antibody-drug conjugate (ADC). Oncotarget.

[16] Goldstein AS, Huang J, Guo C, Garraway IP, Witte ON (2010). Identification of a cell of origin for human prostate cancer. Science.

[17] Goldstein AS, Lawson DA, Cheng D, Sun W, Garraway IP, Witte ON (2008). Trop2 identifies a subpopulation of murine and human prostate basal cells with stem cell characteristics. Proc Natl Acad Sci USA.

[18] King GT, Eaton KD, Beagle BR, Zopf CJ, Wong GY, Krupka HI, Hua SY, Messersmith WA, El-Khoueiry AB (2018). A phase 1, dose-escalation study of PF-06664178, an anti-Trop-2/Aur0101 antibody-drug conjugate in patients with advanced or metastatic solid tumors. Invest New Drugs.

[19] Cardillo TM, Govindan SV, Sharkey RM, Trisal P, Arrojo R, Liu D, Rossi EA, Chang CH, Goldenberg DM (2015). Sacituzumab govitecan (IMMU-132), an anti-trop-2/SN-38 antibody-drug conjugate: Characterization and efficacy in pancreatic, gastric, and other cancers. Bioconjug Chem.

[20] Cardillo TM, Govindan SV, Sharkey RM, Trisal P, Goldenberg DM (2011). Humanized anti-Trop-2 IgG-SN-38 conjugate for effective treatment of diverse epithelial cancers: Preclinical studies in human cancer xenograft models and monkeys. Clin Cancer Res.

[21] Okajima D, Yasuda S, Yokouchi Y, Fujitani T, Sakurai K, Yamaguchi J (2018). Preclinical efficacy studies of DS-1062a, a novel TROP2-targeting antibody-drug conjugate with a novel DNA topoisomerase I inhibitor DXd. J Clinical Oncol.

[22] Itai S, Fujii Y, Nakamura T, Chang YW, Yanaka M, Saidoh N, Handa S, Suzuki H, Harada H, Yamada S (2017). Establishment of CMab-43, a sensitive and specific anti-CD133 monoclonal antibody, for immunohistochemistry. Monoclon Antib Immunodiagn Immunother.

[23] Yamada S, Kaneko MK, Sayama Y, Asano T, Sano M, Yanaka M, Nakamura T, Okamoto S, Handa S, Komatsu Y (2020). Development of novel mouse monoclonal antibodies against human CD19. Monoclon Antib Immunodiagn Immunother.

[24] Furusawa Y, Kaneko MK, Kato Y (2020). Establishment of C20Mab-11, a novel anti-CD20 monoclonal antibody, for the detection of B cells. Oncol Lett.

[25] Yamada S, Itai S, Nakamura T, Yanaka M, Kaneko MK, Kato Y (2018). Detection of high CD44 expression in oral cancers using the novel monoclonal antibody, C(44)Mab-5. Biochem Biophys Rep.

[26] Yamada S, Itai S, Nakamura T, Yanaka M, Chang YW, Suzuki H, Kaneko MK, Kato Y (2018). Monoclonal antibody L(1)Mab-13 detected human PD-L1 in lung cancers. Monoclon Antib Immunodiagn Immunother.

[27] Fujii Y, Kaneko M, Neyazaki M, Nogi T, Kato Y, Takagi J (2014). PA tag: A versatile protein tagging system using a super high affinity antibody against a dodecapeptide derived from human podoplanin. Protein Expr Purif.

[28] Fujii Y, Kaneko MK, Ogasawara S, Yamada S, Yanaka M, Nakamura T, Saidoh N, Yoshida K, Honma R, Kato Y (2017). Development of RAP Tag, a novel tagging system for protein detection and purification. Monoclon Antib Immunodiagn Immunother.

[29] Fujii Y, Kaneko MK, Kato Y (2016). MAP Tag: A novel tagging system for protein purification and detection. Monoclon Antib Immunodiagn Immunother.

[30] Kohler G, Milstein C (1975). Continuous cultures of fused cells secreting antibody of predefined specificity. Nature.

[31] Kato Y, Kaneko MK, Kuno A, Uchiyama N, Amano K, Chiba Y, Hasegawa Y, Hirabayashi J, Narimatsu H, Mishima K, Osawa M (2006). Inhibition of tumor cell-induced platelet aggregation using a novel anti-podoplanin antibody reacting with its platelet-aggregation-stimulating domain. Biochem Biophys Res Commun.

[32] Itai S, Fujii Y, Kaneko MK, Yamada S, Nakamura T, Yanaka M, Saidoh N, Chang YW, Handa S, Takahashi M (2017). H2Mab-77 is a sensitive and specific Anti-HER2 monoclonal antibody against breast cancer. Monoclon Antib Immunodiagn Immunother.

[33] Furusawa Y, Yamada S, Itai S, Nakamura T, Yanaka M, Sano M, Harada H, Fukui M, Kaneko MK, Kato Y (2019). PMab-219: A monoclonal antibody for the immunohistochemical analysis of horse podoplanin. Biochem Biophys Rep.

[34] Furusawa Y, Kaneko MK, Nakamura T, Itai S, Fukui M, Harada H, Yamada S, Kato Y (2019). Establishment of a monoclonal antibody PMab-231 for tiger podoplanin. Monoclon Antib Immunodiagn Immunother.

[35] Furusawa Y, Takei J, Sayama Y, Yamada S, Kaneko MK, Kato Y (2019). Development of an anti-bear podoplanin monoclonal antibody PMab-247 for immunohistochemical analysis. Biochem Biophys Rep.

[36] Furusawa Y, Yamada S, Itai S, Nakamura T, Takei J, Sano M, Harada H, Fukui M, Kaneko MK, Kato Y (2019). Establishment of a monoclonal antibody PMab-233 for immunohistochemical analysis against Tasmanian devil podoplanin. Biochem Biophys Rep.

[37] Polyak MJ, Li H, Shariat N, Deans JP (2008). CD20 homo-oligomers physically associate with the B cell antigen receptor. Dissociation upon receptor engagement and recruitment of phosphoproteins and calmodulin-binding proteins. J Biol Chem.

[38] Li H, Ayer LM, Lytton J, Deans JP (2003). Store-operated cation entry mediated by CD20 in membrane rafts. J Biol Chem.

[39] Kaneko MK, Nakamura T, Honma R, Ogasawara S, Fujii Y, Abe S, Takagi M, Harada H, Suzuki H, Nishioka Y, Kato Y (2017). Development and characterization of anti-glycopeptide monoclonal antibodies against human podoplanin, using glycan-deficient cell lines generated by CRISPR/Cas9 and TALEN. Cancer Med.

[40] Ogasawara S, Kaneko MK, Kato Y (2016). LpMab-19 recognizes sialylated O-Glycan on Thr76 of human podoplanin. Monoclon Antib Immunodiagn Immunother.

[41] van Rij CM, Frielink C, Goldenberg DM, Sharkey RM, Lütje S, McBride WJ, Oyen WJG, Boerman OC (2014). Pretargeted radioimmunotherapy of prostate cancer with an anti-TROP-2×Anti-HSG bispecific antibody and a (177)Lu-labeled peptide. Cancer Biother Radiopharm.

[42] Nishimura T, Mitsunaga M, Sawada R, Saruta M, Kobayashi H, Matsumoto N, Kanke T, Yanai H, Nakamura K (2019). Photoimmunotherapy targeting biliary-pancreatic cancer with humanized anti-TROP2 antibody. Cancer Med.

[43] Zhao W, Jia L, Zhang M, Huang X, Qian P, Tang Q, Zhu J, Feng Z (2019). The killing effect of novel bi-specific Trop2/PD-L1 CAR-T cell targeted gastric cancer. Am J Cancer Res.

