# Supplementary material for: Development and characterization of TrMab-6, a novel anti-TROP2 monoclonal antibody for antigen detection in breast cancer
Source: Mol Med Rep. 2020 Nov 25;23(2):92. doi: 10.3892/mmr.2020.11731 (PMC7723163; doi:10.3892/mmr.2020.11731)
Supplement: Supporting Data [file Supplementary_Data.pdf]

Figure S1. The comparison of amino acid sequences at the hinge region of mouse IgGs, and the confirmation of transfection for TROP2 using western blot analysis. (A) The hinge sequence of TrMab-6 was identical to that of mouse IgG_{2b}. (B) The cell lysates (10 μg) of CHO-K1, CHO/TROP2-PA, MCF7, BINDS-29 (MCF7/TROP2-KO), BT-474, Lec1/TROP2, Lec2/TROP2 and Lec8/TROP2 were subjected to SDS-PAGE and transferred onto the PVDF membranes. The membranes were incubated with an anti-PA mAb (NZ-1) and an anti-β-actin mAb (AC-15), followed by secondary antibodies. *Identical amino acid among mouse IgG subclasses. TROP2, Trophoblast cell-surface antigen 2; mAb, monoclonal antibody.

A

TrMab-6	EPSGPISTINPCPPCKECHKCPA
Mouse IgG_{2b}	EPSGPISTINPCPPCKECHKCPA
Mouse IgG_{2a}	EPRGP--TIKPCPPC----KCPA
Mouse IgG_3	EPRIP----KPSTPPGS--SCPP
Mouse IgG_1	VPRDC-----GCKPC----ICTV
	` *        . *     *.`

B

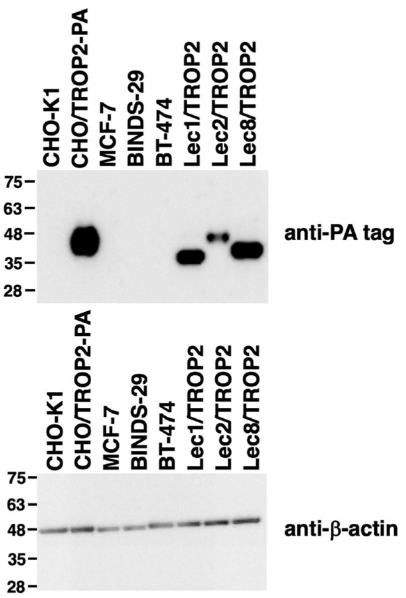

Figure S2. Flow cytometric detection of TROP2 using TrMab-6 and EPR20043. MCF7 (A and B), BINDS-29 (MCF7/TROP2-KO) (C and D), and BT-474 (E and F) cells were incubated with TrMab-6 (1 μg/ml; red line) (A, C and E), EPR20043 (1/60 dilution; blue line) (B, D and F) or 0.1% BSA in PBS (gray) for 30 min, followed by Alexa Fluor 488-conjugated secondary antibodies. Fluorescence data were collected using a cell analyzer. TROP2, Trophoblast cell-surface antigen 2.

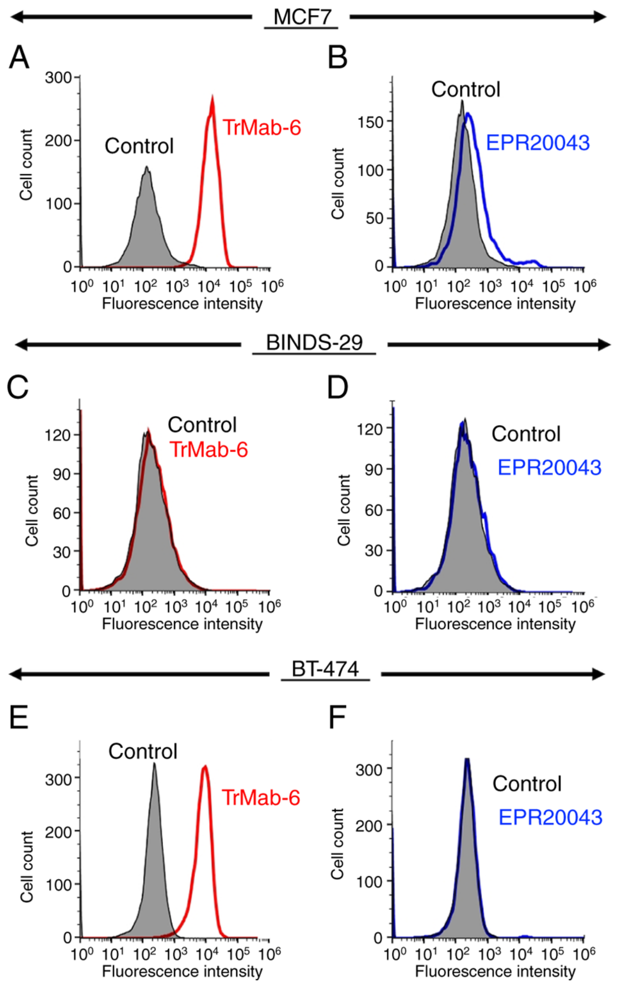

Figure S3. Detection of TROP2 with TrMab-6 and EPR20043 by western blot analysis. The cell lysates (10 μg) of MCF7, BINDS-29 (MCF7/TROP2-KO), and BT-474 were subjected to SDS-PAGE and transferred onto the PVDF membranes. The membranes were incubated with (A) TrMab-6 (5 μg/ml), (B) EPR20043 (1:2,000 dilution) and an (C) anti-β-actin mAb (AC-15; 1 μg/ml), followed by secondary antibodies. TROP2, Trophoblast cell-surface antigen 2; mAb, monoclonal antibody.

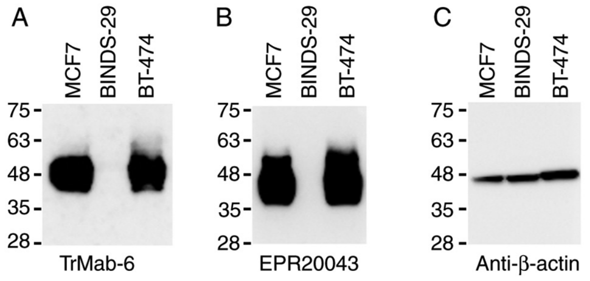

Table SI. Commercially available anti-human TROP2 mAbs.

Clone	Company	Species	Isotype	Immunogen	Applications		
					Flow cytometry	Immunohistochemistry	Western blot
NY18	BioLegend, Inc.	Mouse	IgG_{2a}	PK-1 (Human)	+	-	-
001	Sino Biological, Inc.	Rabbit	IgG	Protein	+	-	-
TACSTD2/2153	Biorbyt Ltd	Mouse	IgG_1	Protein	-	+	-
LS-C178765	LifeSpan BioSciences, Inc.	Mouse	IgG_1	Protein	-	+	-
LS-C489658	LifeSpan BioSciences, Inc.	Mouse	-	Protein	-	+	-
TA310025	Origene Technologies, Inc.	Mouse	IgG_1	Human choriocarcinoma cell line BeWo	-	+	-
8C6C1D7	Sino Biological, Inc.	Mouse	IgG_1	Protein	-	+	-
M005	Abcam	Mouse	IgG_1	Carcinoma cell line A431	-	-	+
H00004070-K	Abnova	Rabbit	IgG	Peptide	-	-	+
D1W5W	Cell Signaling Technology, Inc.	Rabbit	IgG	Peptide	-	-	+
162-46.2	Abcam	Mouse	IgG_1	Human choriocarcinoma cell line BeWo	+	+	-
01	Enzo Life Sciences	Mouse	IgG_1	Protein	+	+	-
LS-C489657	LifeSpan BioSciences, Inc.	Mouse	IgG	Protein	+	+	-
1	Thermo Fisher Scientific, Inc.	Rabbit	IgG	Protein	+	+	-
49D6	GeneTex, Inc.	Mouse	IgG_2	Protein	+	-	+
SP294	Abcam	Rabbit	IgG	Peptide	-	+	+
SP295	Abcam	Rabbit	IgG	Peptide	-	+	+
TACSTD2/2152	Biorbyt Ltd	Mouse	IgG_1	Protein	-	+	+
TACSTD2/2151	Biorbyt Ltd	Mouse	IgG_{2b}	Protein	-	+	+
TCSR2-1	Biorbyt Ltd	Mouse	IgG_1	Protein	-	+	+
EPR20043	Abcam	Rabbit	IgG	Peptide	+ (intracellular)	+	+
SP293	Abcam	Rabbit	IgG	Peptide	+ (intracellular)	+	+

TROP2, Trophoblast cell-surface antigen 2; mAb, monoclonal antibody.
